# Genome-wide characterization, phylogenetic and expression analysis of *Galectin* gene family in Golden pompano *Trachinotus ovatus*

**DOI:** 10.3389/fimmu.2024.1452609

**Published:** 2024-07-18

**Authors:** Jin-Min Pan, Yu Liang, Ke-Cheng Zhu, Hua-Yang Guo, Bao-Suo Liu, Nan Zhang, Lin Xian, Teng-Fei Zhu, Dian-Chang Zhang

**Affiliations:** ^1^ Key Laboratory of South China Sea Fishery Resources Exploitation and Utilization, Ministry of Agriculture and Rural Affairs; South China Sea Fisheries Research Institute, Chinese Academy of Fishery Sciences, Guangzhou, Guangdong, China; ^2^ Guangdong Provincial Engineer Technology Research Center of Marine Biological Seed Industry, Guangzhou, Guangdong, China; ^3^ Sanya Tropical Fisheries Research Institute, Sanya, China; ^4^ Shenzhen Base of South China Sea Fisheries Research Institute, Chinese Academy of Fishery Sciences, Shenzhen, Guangdong, China

**Keywords:** Galectins, Golden pompano, subcellular localization, protein function, bacterial agglutination

## Abstract

Galectins (Gals) are a type of S-type lectin that are widespread and evolutionarily conserved among metazoans, and can act as pattern recognition receptors (PRRs) to recognize pathogen-associated molecular patterns (PAMPs). In this study, *10 Gals* (*ToGals*) were identified in the Golden pompano (*Trachinotus ovatus*), and their conserved domains, motifs, and collinearity relationships were analyzed. The expression of *ToGals* was regulated following infection to *Cryptocaryon irritans* and *Streptococcus agalactiae*, indicating that *ToGals* participate in immune responses against microbial pathogens. Further analysis was conducted on one important member, Galectin-3, subcellular localization showing that ToGal-3like protein is expressed both in the nucleus and cytoplasm. Recombinant protein obtained through prokaryotic expression showed that rToGal-3like can agglutinate red blood cells of rabbit, carp and golden pompano and also agglutinate and kill *Staphylococcus aureus, Bacillus subtilis, Vibrio vulnificus, S. agalactiae, Pseudomonas aeruginosa*, and *Aeromonas hydrophila*. This study lays the foundation for further research on the immune roles of *Gals* in teleosts.

## 1 Introduction

Teleost, as aquatic vertebrates, possess a relatively lower complexity compared to mammals and primarily defend against the myriad of pathogens in their environment through innate immunity. Renowned for its rapid response, this form of immunity includes physical barriers such as skin, mucus, and gills, which constitute the first line of defense in the teleost immune system (1). Research on teleosts has revealed that their mucus contains a combination of agglutinins, lysozymes, antimicrobial peptides, and complement proteins, all of which work together to directly neutralize pathogens (2). When pathogens encounter these physical barriers, Pattern Recognition Receptors (PRRs) recognize Pathogen-Associated Molecular Patterns (PAMPs) on the pathogens, thereby activating the innate immune response (3).

lectins not only play a crucial role within physical barriers but are also key components of PRRs. Initially, animal agglutinins were classified into calcium-dependent C-type lectins and S-type lectins Galectins are a part of the S-type lectins (4). Based on their molecular structure, galectins can be divided into three types: proto-type, which contains only one Carbohydrate Recognition Domain (CRD); tandem repeat type, which features two homologous CRDs at both the N and C termini; and chimeric type like Galectin-3 (5). Galectin-related protein (GRP) has similar structural domains and is often analyzed together with Gals (6).

*Trachinotus ovatus*, commonly known as golden pompano, is an economically important fish species in the South China Sea. In 2023, it ranked second in marine fish production in China, renowned for its firm flesh and rapid growth (7). In previous studies on Golden pompano, extensive research was conducted on PRRs, such as the classic Toll-like Receptor (TLRs) and NOD-like Receptor (NLRs) (8–10). as well as on proteins with antimicrobial functions like antimicrobial peptides (11, 12). However, these PRRs-Galectins, which also have the antimicrobial functions, have not yet been identified in Golden pompano.

Under high-density aquaculture conditions, outbreaks of bacterial and parasitic infections are common. Among these pathogens, *Streptococcus agalactiae* and *Cryptocaryon irritans* pose significant threats to economically important fish species. Teleosts, whether marine or freshwater species, typically exhibit enlarged spleens and livers and accumulate large amounts of fluid in the abdominal cavity following infection with *S. agalactiae* (13). For Golden pompano, *S. agalactiae* is one of the most significant bacterial diseases (14). *C. irritans* is a ciliated obligate parasite predominantly infesting the outer body surfaces of fish, such as gills, skin, and fins, causing illness in marine teleosts. Commonly referred to as white spot disease due to its visually detectable white cysts, *C. irritans* can inflict substantial damage on host organs. This damage includes respiratory difficulties, abnormal behavior, mechanical injuries, and susceptibility to secondary bacterial infections (15). More seriously, the mortality rate for golden pompano infected with *C. irritans* can reach 100% (16).

This study has identified the *T. ovatus galectins* (*ToGals*) in whole genome wide. The conserved domain, collinearity and evolutionary relationship were analyzed. Moreover, we conducted an infection test for *S. agalactiae* and *C. irritans*, analyzing the differences of expression patterns following infection. Additionally, we conducted subcellular localization of a member of *ToGals* - *ToGal3like*, in the Golden pompano muscle cell line (GPM). At last, the recombination ToGal3like protein was also expressed using a prokaryotic expression system, and its hemagglutination and bacterial agglutination activities were analyzed. The objective of this study is to provide guidance for the prevention and control of pathogenic microbes in *T. ovatus*, and to further lay a foundation for research into the functions of Gals in *T. ovatus* and other teleost species.

## 2 Materials and methods

### 2.1 The identification of *ToGals*

To identify the *ToGals*, amino acid sequences of Galectins from closely related species, the Yellowtail kingfish and the Great amberjack, were initially download from the Ensembl database. Based on these sequences, the genome of *T. ovatus* was searched using TblastN (https://blast.ncbi.nlm.nih.gov/) (e-value of <1e-10), revealing several candidate genes. Additionally, Hidden Markov Models (HMM) of the Gal-lectin domain were retrieved from the Pfam database (http://pfam.sanger.ac.uk/). Subsequently, genes containing the Gal-lect domain were screened using the HMM searcher (*P* < 0.05) (17). Ultimately, by integrating both methods, the candidate *ToGals* genes have been identified.

### 2.2 Molecular characterization and collinearity analysis of *ToGals*

To enhance the understanding of *ToGals*, the full mRNA and coding sequences (CDS) for these genes were retrieved using the GTF annotations of the *T. ovatus* genome (18). Protein sequences were analyzed using ProtParam on the ExPASy (https://expassy.org/protparam/) to determine the molecular weights (MW) and isoelectric points (PI) of the ToGals. Domains within the proteins were defined using a comprehensive search on the NCBI-CDD (https://www.ncbi.nlm.nih.gov/Structure/cdd/cdd.shtml). Using Meme for Linux (https://meme-suite.org/meme/) (19), we identified five key motifs across *ToGals* mRNA. Comparative analysis was conducted to examine the variances in the total *Gals* copy numbers between *T. ovatus* and other species. Finally, we analyzed the collinearity of *ToGals* using McscanX (20) and visualized it with Circos (21).

### 2.3 Phylogenetic analysis of ToGals

For the phylogenetic analysis of ToGals, amino acid sequences of galectins from *sapiens, Mus musculus, Danio rerio, Scophthalmus maximus, Takifugu rubripes*, and *Pseudoplatystoma corruscans*, along with the sequences of ToGals, were downloaded from NCBI to create a galectin amino acid library. The sequences were aligned using MAFFT (https://mafft.cbrc.jp/alignment/software/). A maximum likelihood (ML) tree was then constructed using FastTree. The final visualization of the phylogenetic tree was done using Chiplot (https://www.chiplot.online/).

### 2.4 Pathogens infection experiment

The *T. ovatus* used in this experiment were all sourced from marine cages in DaPeng Bay, Shenzhen, and were transported to an experimental concrete pond for acclimatization for half a month prior to the experiment, with a salinity of 15‰, pH 8.0 ± 0.2, and water temperature at 28.5 ± 1.5°C. The Committee of the South China Sea Fisheries Research Institute, Chinese Academy of Fisheries Sciences (no. SCSFRI96-254), approved the animal protocols, and all experiments were performed under the applicable standards.

#### 2.4.1 *S. agalactiae* infection experiment

*S. iniae* was identified from infected *T. ovatus* in an aquaculture pond in Dapeng, Shenzhen. After purification and identification, 100 mL of the bacterial suspension were spread on BHI agar plates and incubated overnight at 28°C. A bacterial colony was then selected and cultured in BHI liquid medium on a shaker to expand. Following 16S rDNA sequencing performed by Guangzhou Ruibo Biotechnology, the bacteria were confirmed to be *S. iniae* via BLAST. Prior to infection, *S. iniae* was cultured for 24 hours in BHI liquid medium under specific conditions (28°C and 140 × g). The sediment was separated by centrifugation at 6200 × g for 8 m, then diluted and spread on plates at five different concentrations, ranging from 1.0 × 10^6^ to 1.0 × 10^10^ CFU/ml. The concentrations were quantified by measuring colony-forming units per milliliter. The 120-hour lethal dose 50 (LD50) was found to be 2.0 × 10^7^ CFU per *T. ovatus*, obtained by dilution in PBS (22). In the actual experiment, six tanks each containing 140 L of fresh seawater were used. Three hundred healthy *T. ovatus* were evenly and randomly divided into control and experimental groups, with three replicates each. The fish in the experimental group were injected with a bacterial mixture of 2.0 × 107 CFU/fish (volume of 200 mL), while the control group received an injection of 200 mL of sterile PBS. Samples were taken immediately after infection, and then 48 and 96 hours later. At each time point, samples were collected from the liver, spleen, and kidneys of nine randomly selected fish from each group. After anesthetizing the *T. ovatus* with 40 mg/L MS-222, samples from three *T. ovatus* were pooled into one sample. The samples were then rapidly frozen in liquid nitrogen and stored at -80°C.

#### 2.4.2 *C. irritans* infection experiment

*C. irritans* were sourced from *T. ovatus* infected at the Dapeng bay. The organism was identified as *C. irritans* via microscopic examination and was further propagated using *T. ovatus* as host organisms. Prior to the infection experiments, skin samples from nine unexposed, healthy *T. ovatus* were collected to serve as a control group (BFS).

The infection method for *C. irrita* was carried out according to Dan et al. (23). Preliminary studies proceeded to establish the LD50 of *C. irritans* infection of *T. ovatus* (24). This involved placing the fish in 0.5 m^3^ plastic cylinders containing water at a density of 8000 tomite/L. Following a brief 10-second infection to this infectious dose, the fish were relocated to a concrete holding tank. Skin samples, including those from areas where *C. irritans* trophonts attached areas (TAS) and adjacent areas (NRS), were collected 48 h after infection. The samples were rapidly frozen in liquid nitrogen and stored at -80°C, with three replicates for each group. These specimens were then sent to Novogene (TJ, China) for RNA sequencing analysis.

#### 2.4.3 Analysis of *ToGals* expression levels

Using RNA-seq data, we computed FPKM values for each experimental group and focused our analysis on the identified *ToGals* to examine their expression variations among different groups. To pinpoint *ToGals* that exhibited statistically significant changes in expression, we set a criterion of |log2(FoldChange)| > 1, with a *P*adj no greater than 0.05. We also applied the Benjamini–Hochberg correction to the *P*-values to reduce the risk of false positives. For effective presentation, we visualized the expression data using Chiplot (https://www.chiplot.online/).

### 2.5 Subcellular localization assay

Primers F 5’ CGGCAGCCATATGGATCTCTCAGA 3’ and R 5’ TGGTGGTGCTCGAGTTAGGGCAAG 3’, with the underlined positions indicating the restriction sites, were used to amplify the cDNA fragment *ToGal-3like* ORF for subcellular localization. Subsequently, the amplified ORF was cloned into the pEGFP-N3 vector, which had been digested with the restriction enzymes Xho I and BamH I, to construct pEGFP-N3-ToGal3like. For subcellular localization, the Golden pompano Muscle cell line (GPM) from our research group has been used (19). The specific steps are as follows: the GPM were revived and passaged three times to ensure stability before being cultured in a six-well plate. pEGFP-N3 and pEGFP-N3-ToGal3like were each transfected into three wells using Lipo8000 (Beyotime, Shanghai, China). Twenty-four hours later, observations were made using the inverted fluorescence microscope (Leica, DMi8, Germany).

### 2.6 Prokaryotic expression and purification

To express the ToGal-3like protein in prokaryotic cells, the plasmid (pet28a) was ligated with the ORF of *ToGal-3like* transformed into Rosetta (DE3) Super Competent Cells. Once the bacterial culture reached an optical density (OD) of 0.8, IPTG was added to a final concentration of 0.1 mM, followed by incubation at 37°C with shaking for 4 hours. After centrifugation, the pellet was resuspended in 40 μL of 1×loading buffer. A 10μL aliquot was then subjected to SDS-PAGE for analysis. Once the appropriate expression was confirmed, large-scale expression was initiated. In brief, the IPTG-induced bacteria were resuspended in NTA-0 buffer, treated with lysozyme, and incubated in an ice bath for 30 minutes. The lysed bacteria were then subjected to ultrasonication, followed by centrifugation to separate the supernatant and pellet, which were further analyzed by SDS-PAGE. The remaining sample was stored at 4°C for subsequent use. For the final step of protein purification, the supernatant was passed through a Ni-NTA column (Invitrogen™), and the purified rToGal-3like was analyzed again using SDS-PAGE.

### 2.7 Hemagglutination assay

Blood was collected from rabbits, carp (freshwater fish) and golden pompano (male: female = 1:1). After centrifugation, the blood samples were washed three times using PBS and then resuspended to produce a 2% RBCs (red blood cells) solution. To this, either 100 μl of 2% RBCs solution was mixed with 250 μl/ml rToGal-8 solution or 100 μl of a solution with a final concentration of 10 mM CaCl_2_ and 250 μl/ml rToGal-3like. For the control group, 6 x HIS tag protein was used under the same conditions. After incubation for one hour, the samples were observed under an optical microscope. Each group was replicated three times.

### 2.8 Bacterial agglutination/killing assay

In a nutshell, strains of *Staphylococcus aureus, Bacillus subtilis, Vibrio vulnificus, S. agalactiae, Pseudomonas aeruginosa*, and *Aeromonas hydrophila* were isolated and cultured on agar plates. Following the selection of individual colonies, sequencing was carried out for identification. Post-identification, they were further cultivated in LB liquid medium until they reached the logarithmic growth phase. Bacterial cells were harvested by centrifugation at 6000 × g for 5 minutes and subsequently resuspended in physiological saline. Treatment began with a starting concentration of 1mg/ml of rToGal-3like, which was serially diluted in a twofold manner. The rToGal-3like solution was then combined with the bacterial suspension and incubated at room temperature for 2 h. Following this, the Live/Dead^®^ BacLight™ Bacterial Viability Kit (Invitrogen) was introduced to the mixture. After blending the dye with an equal volume of the bacterial and protein solution, it was kept in dark conditions for an incubation period of 15 minutes. The same concentration BSA solution served as the control group. Observations and image captures were carried out using a Leica inverted fluorescence microscope (DMI8). Each group was replicated three times.

## 3 Result

### 3.1 The identification of *ToGals*

Using homologous genes from other species and Hidden Markov Models of the CRD domains, this study identified the longest transcripts and amino acid sequences of 10 *ToGals* members (**Table 1**) through screening with BLAST and HMM searcher software. Their mRNA lengths range from 917 bp (*Galectin-2b*) to 5957 bp (*GRPb*), with the longest being *GRPb* and the shortest being Galectin-2b. The range of isoelectric points is from 5.00 (*Galectin-3like*) to 9.90 (*GRPa*).

**Table 1:** Characteristics of the Galectin gene family in the genome of Golden pompano.

Gene	Chromosome	Start Position	End Position	mRNA Length	Isoelectric Point	Molecular Weight (Da)
*Galectin-2a*	LG16	2860501	2865154	1344	6.28	14675.63
*Galectin-3*	LG19	2993966	2999406	1993	8.55	28468.75
*Galectin-8*	LG13	7394829	7400881	3303	8.88	34724.04
*GRPc*	LG18	1501724	1507915	1262	7.75	16092.73
*Galectin-9*	LG19	443349	451667	1869	9.10	36808.43
*GRPb*	LG6	29869166	29880510	5957	5.39	20065.78
*GRPa*	LG9	28489735	28497664	2454	9.90	20145.16
*Galectin-2b*	LG9	26228502	26232545	917	5.22	20662.39
*Galectin-3like*	LG18	3514902	3520103	1582	5.00	16197.62
*Galectin-4*	LG5	25144600	25150551	990	6.51	26488.42

### 3.2 Copy numbers of *Gals*

**Figure 1:**
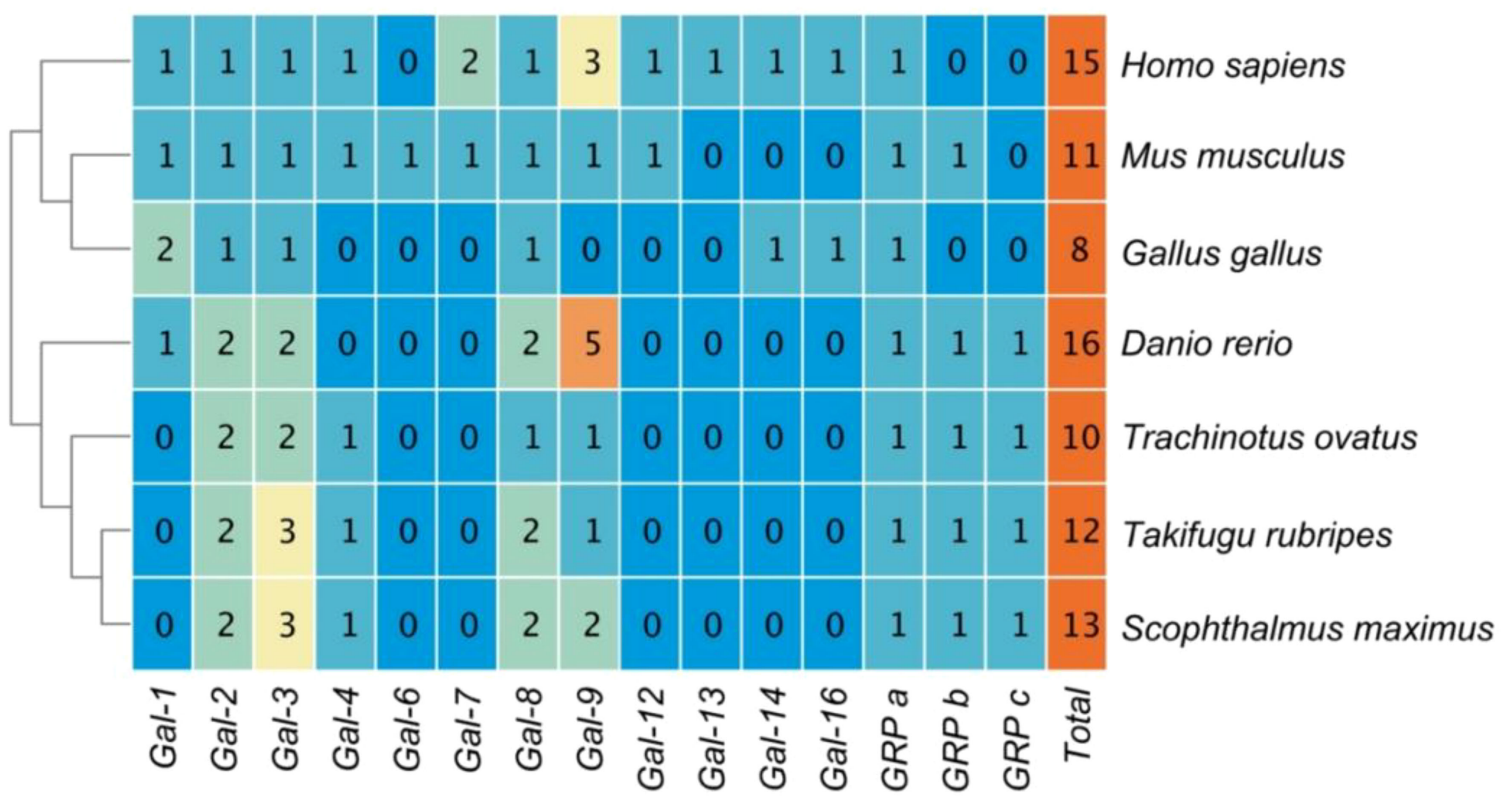
The copy numbers of Galectins in the genomes of several representative model organisms.

Subsequently, this study tallied the copy numbers of *Galectin* members in Golden pompano, humans, mice, chickens, zebrafish, pufferfish, and turbot (**Figure 1**). Overall, from fish to mammals and birds, the number of Galectins is similar, ranging from 8 (in chickens) to 16 (in zebrafish), with humans also possessing 15 Galectins. *Galectin-7* and *Galectin-6* are exclusive to mammals and can be found in teleosts where *GRP* has three copies.

### 3.3 Motif and domain of *ToGals*

**Figure 2:**
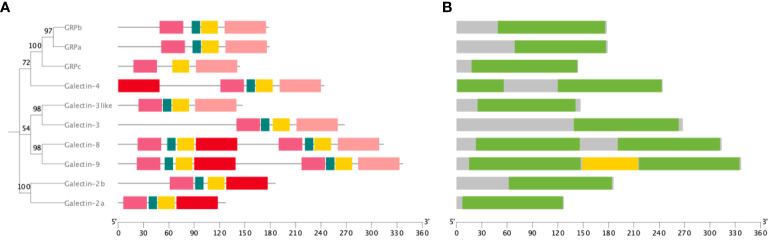
Motif and domain of *ToGals*. **(A)** Motif of *ToGals*. 5 conserved motifs are established by MEME database and represented by 5 different colors.; **(B)** Main domains in *ToGals*.

*ToGals* motifs and domains have been visualized (**Figure 2**), revealing that they all possess Gal-bind lectin domains (**Figure 2B**). Among these, three GRPs and two Galectins - *Galectin-2* and *Galectin-3* - each have one Gal-bind lectin domain. *Galectin-8* and *Galectin-9*, however, each have two such domains. The N-terminal Gal-bind lectins correspond to Motifs 1, 2, 3, and 5, while the C-terminal Gal-bind lectins correspond to Motifs 1, 2, 3, and 4 (**Figure 2A**).

### 3.4 Chromosome localization and collinearity analysis

**Figure 3:**
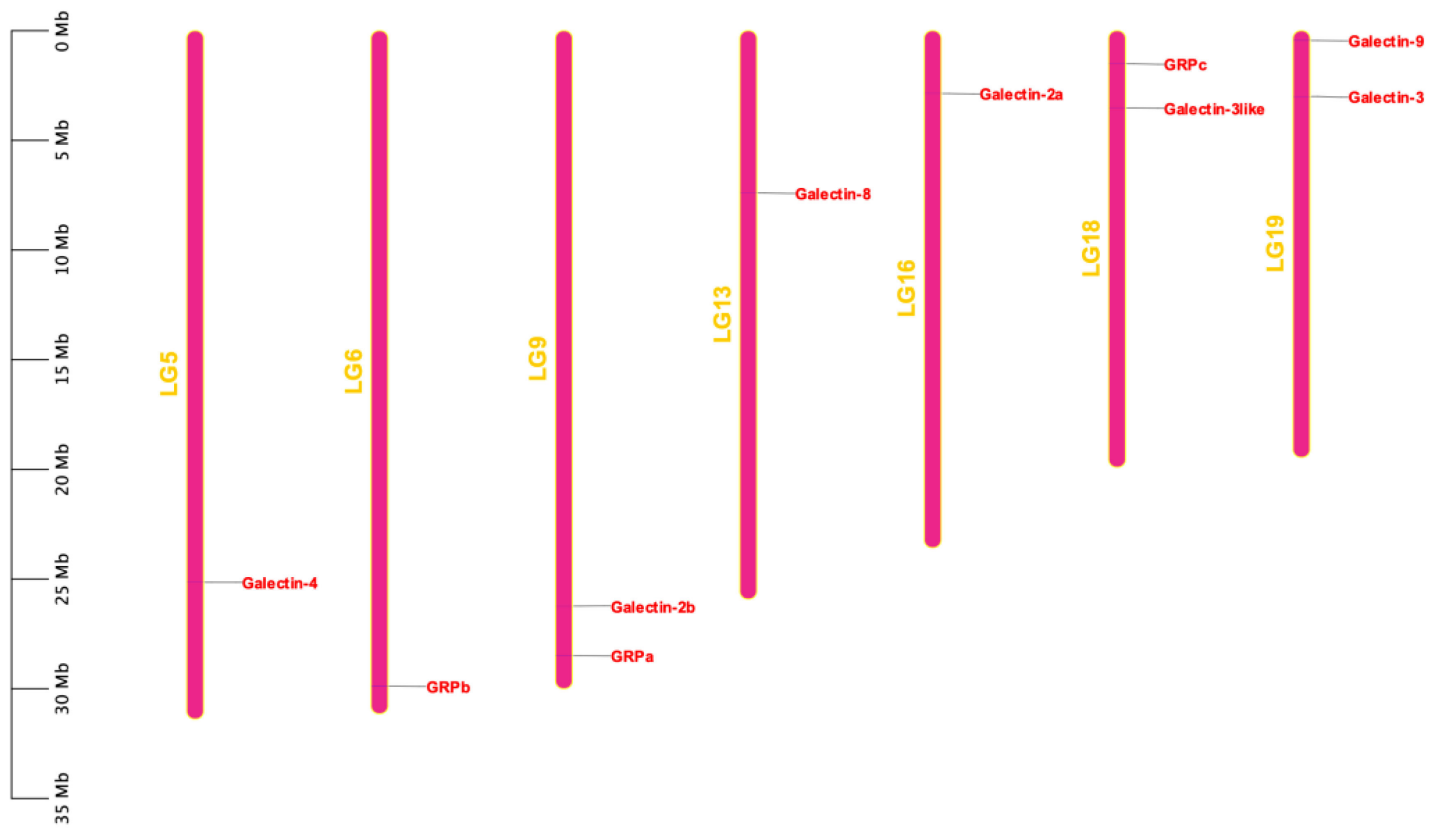
Chromosomal locations of the *ToGals*. The location of *ToGals* in the genome is indicated by a red line and a red label, The size of a chromosome is represented by its relative length.

**Figure 4:**
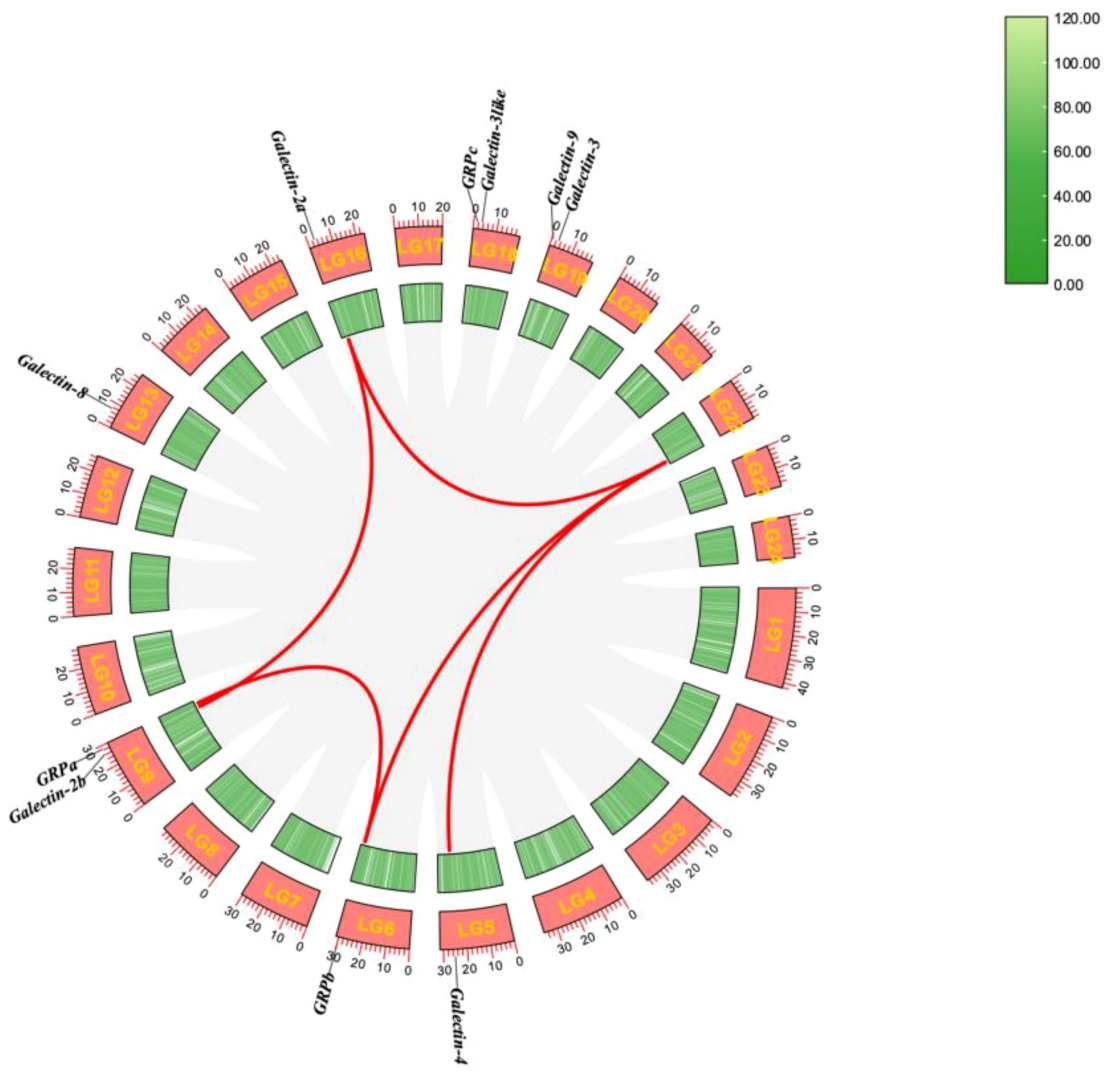
Synteny analysis of the *ToGals* genes in Golden pompano. Gray lines represent all synteny blocks in the Golden pompano genome. Tandem and segmental duplicates are exhibited with red lines.

The 10 *ToGals* were localized on 7 chromosomes. *Galectin-4* is located on LG5, and *GRPb* on LG6. LG9 contains both *Galectin-2b* and *GRPb*; *Galectin-8* is located on LG13, and *Galectin-2a* on LG16. *GRPc* and *Galectin-3like* are positioned on LG18, while *Galectin-8* and *Galectin-3* are on LG19 (**Figure 3**). Subsequently, the analysis of collinearity within *ToGals* was conducted. Five pairs of collinear relationships were identified. It was observed that within *ToGals*, *Galectin-2a* and *Galectin-2b*, as well as *GRPa* and *GRPb*, exhibit collinearity. Moreover, Galectin-4, *GRPb*, and *Galectin-2a* all displayed collinearity with a region on LG22 (**Figure 4**).

### 3.5 Evolutionary relationships analysis

**Figure 5:**
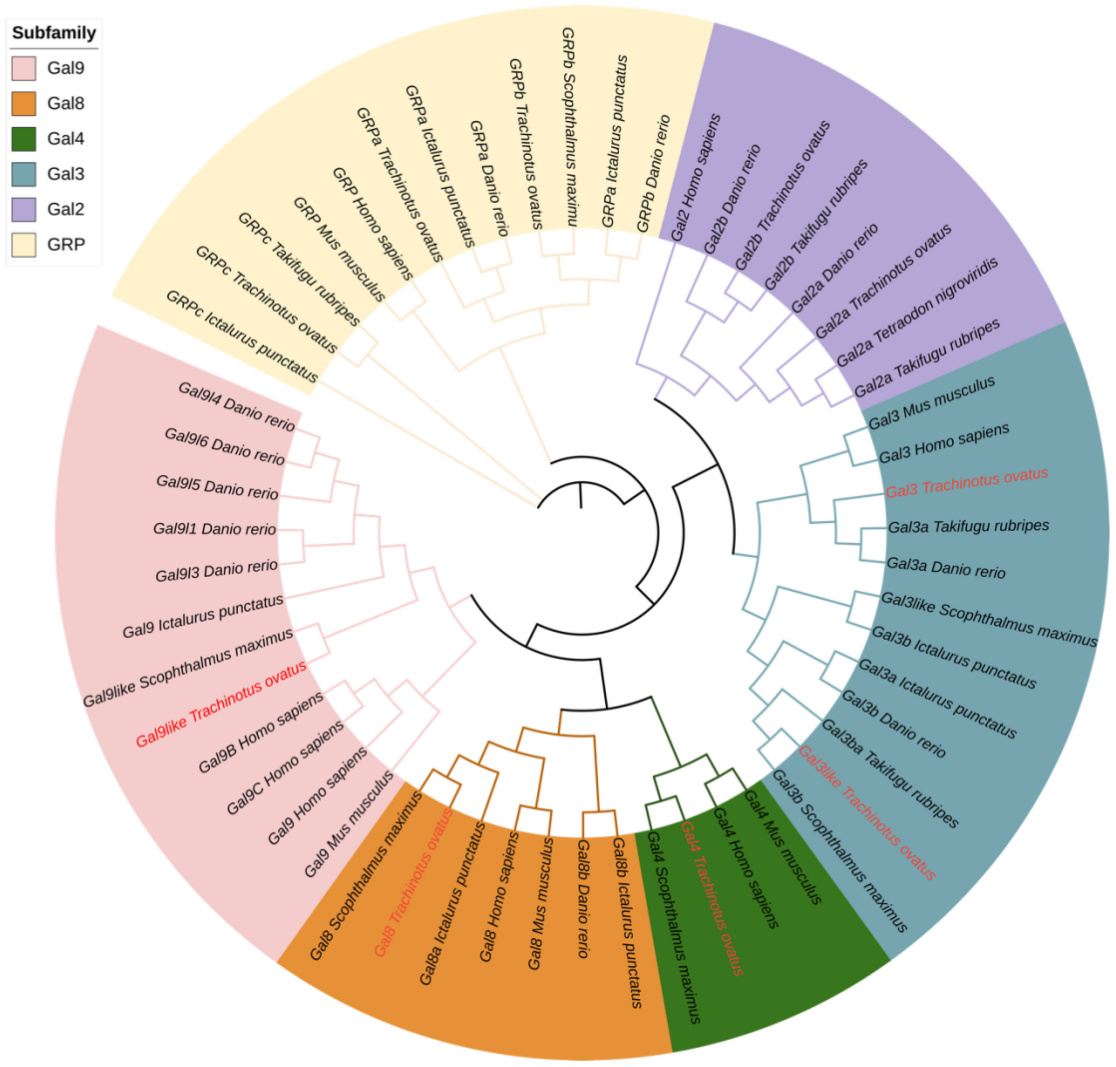
Phylogenetic tree analysis of Gals from different species. The phylogenetic tree was divided into six groups, each with a different color. ToGals labs were marked in red.

Phylogenetic analysis was conducted using FastTree (**Figure 5**). Initially, ToGals grouped together with the direct orthologues from other teleost fish, while human and mouse Galectins formed another distinct clade. Subsequently, within the subfamilies of Galectins, each grouped into a separate branch. The Gal-8, Gal-9, and Gal-4 subfamilies, which are tandem repeat-type galectins, clustered together. Gal-2a and Gal-2b, which are prototypical galectins, formed another branch. Outgroups comprised GRP, GRPa, GRPb, and GRPc, each forming their own branch. This tree aligns with the traditional evolutionary relationships and the evolutionary relations among subfamilies.

### 3.6 Expression of ToGals after pathogens infection

**Figure 6:**
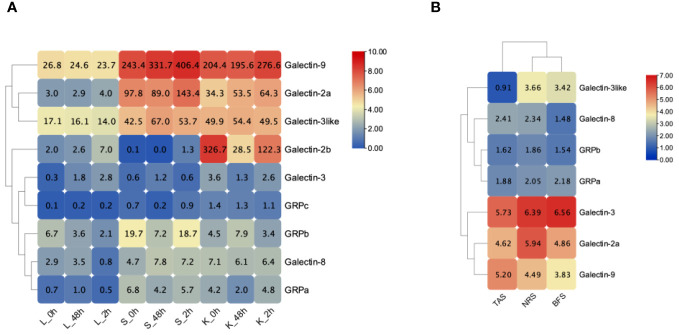
Expression pattern of *ToGals* after infection after pathogens. **(A)**, *S. agalactiae* infection after 0, 48, 96 h in Liver (Li), spleen (Sp) and kidney (Ki); **(B)**, Expression pattern of the skin before infection of *C. irritans* (BFS), the areas containing the trophont attached skin (TAS) and nearby region skin (NRS). The numerical representation in heat map means FPKM.

We analyzed the expression levels of *ToGals* in the spleen, liver, and kidneys following infection with *S. iniae* (**Figure 6A**). The results showed that overall, at all three time points, the expression levels of *Galectin-9*, *Galectin-2a*, and *Galectin-3like* were relatively high across all three tissues. Conversely, *GRPc* and *Galectin-3* exhibited lower expression levels. Interestingly, *Galectin-2b* had lower expression levels in the liver and spleen, but high levels in the kidneys, with a trend of initial decrease followed by an increase from 0h to 96h. Overall, the expression levels of *ToGals* in the liver were lower than in the spleen and kidneys.

Following infection with *C. irritans*, *ToGals* displayed different expression patterns (**Figure 6B**). In various tissues, *Galectin-3*, *Galectin-2a* and *Galectin-9* generally had higher expression levels, while *GRPa* was lower. They also showed varying trends; for *Galectin-9* and *Galectin-8*, the highest expression levels were observed in TAS, whereas other members had higher expression in NRS or BFS.

### 3.7 Subcellular localization of the ToGal-3like

**Figure 7:**
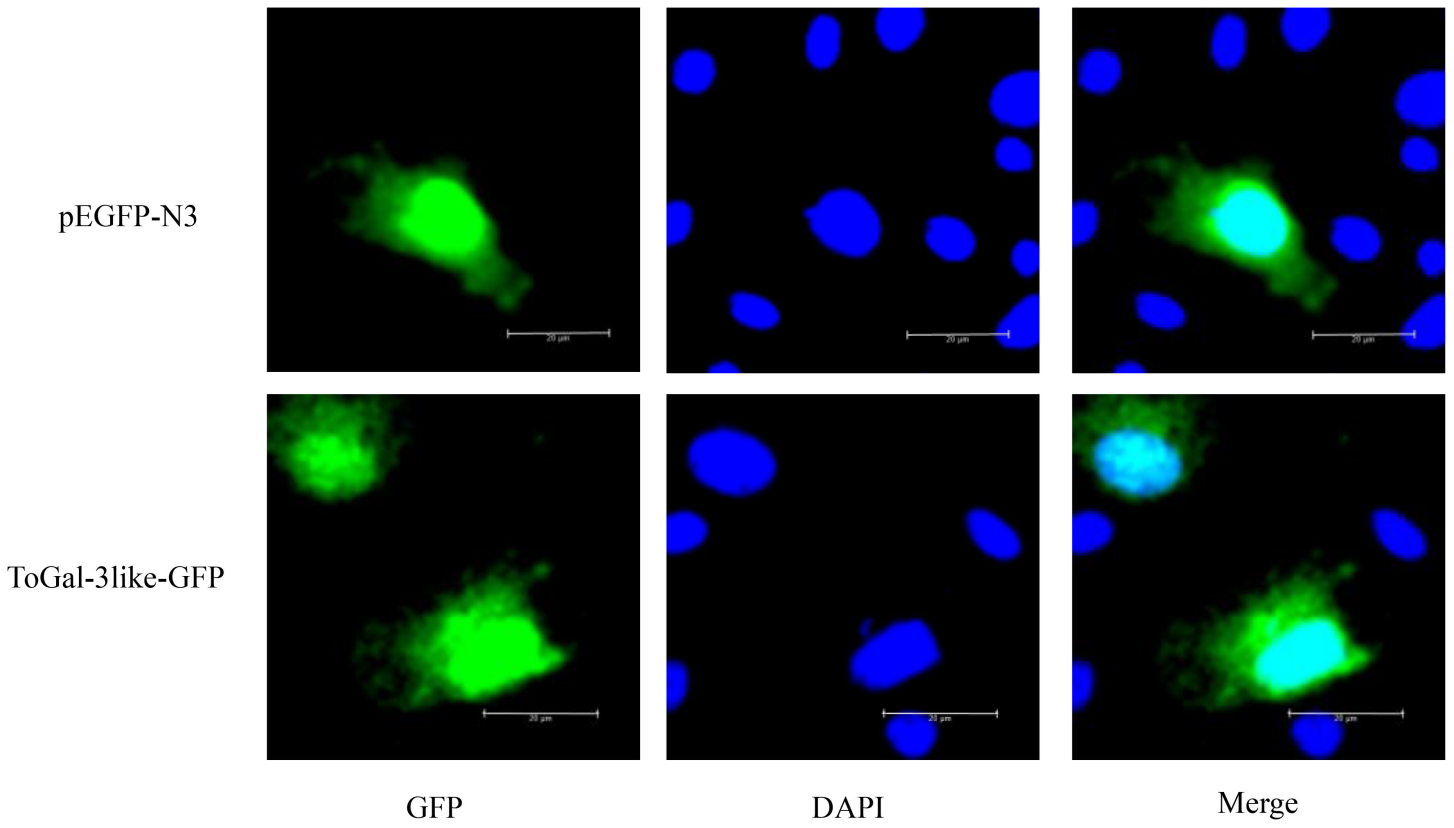
The subcellular localization of ToGal-3like in GPM cells. GPM cells were transfected with pEGFP-N3-ToGal3like, pEGFP-N3. After 24 h, the cells were fixed and the nuclei stained with 4, 6-diamidino-2-phenylindole (DAPI). The left green fluorescence protein panels are pEGFP-N3 and ToGal-3like fusion protein and GFP expression profile under fluorescence, the middle DAPI panels are the cell nucleus stained with DAPI, while the right panels are the combined images of pEGFP-N3, ToGal-3like fusion proteins and GFP with cell nucleus. Bar = 20 μm.

To study the subcellular localization of the ToGal-3like protein, the recombinant plasmid pEGFP-N3-ToGal3like was constructed and transfected into GPM (**Figure 7**), after transfection and DAPI staining, all plasmids expressed green fluorescence overall, while the nuclei exhibited blue fluorescence. ToGal3like was distributed both in the cytoplasm and the nucleus.

### 3.8 Prokaryotic expression and purification of ToGal-3like

Successfully identified recombinant positive clones using antibiotic selection, where clones resistant to kanamycin were matched with ToGal-3like, confirmed by plasmid sequencing. After small-scale expression and IPTG induction, SDS-PAGE analysis showed effective expression of rToGal-3like in the *E. coli* system (**Supplementary Figure 1A**). For large-scale expression, the selected positive clones were cultured, lysed by sonication, and the resultant rToGal-3like (including His tag) displayed the expected MW of approximately 33.48 kDa on SDS-PAGE (**Supplementary Figure 1B**). At last, we purified a total of 500ug/ml of rToGal-3like with 90% purity using Ni-NTA affinity chromatography from the concentrated supernatant, which MW was then verified on SDS-PAGE (**Supplementary Figure 1C**).

### 3.9 Hemagglutination and sugar inhibition assays

**Figure 8:**
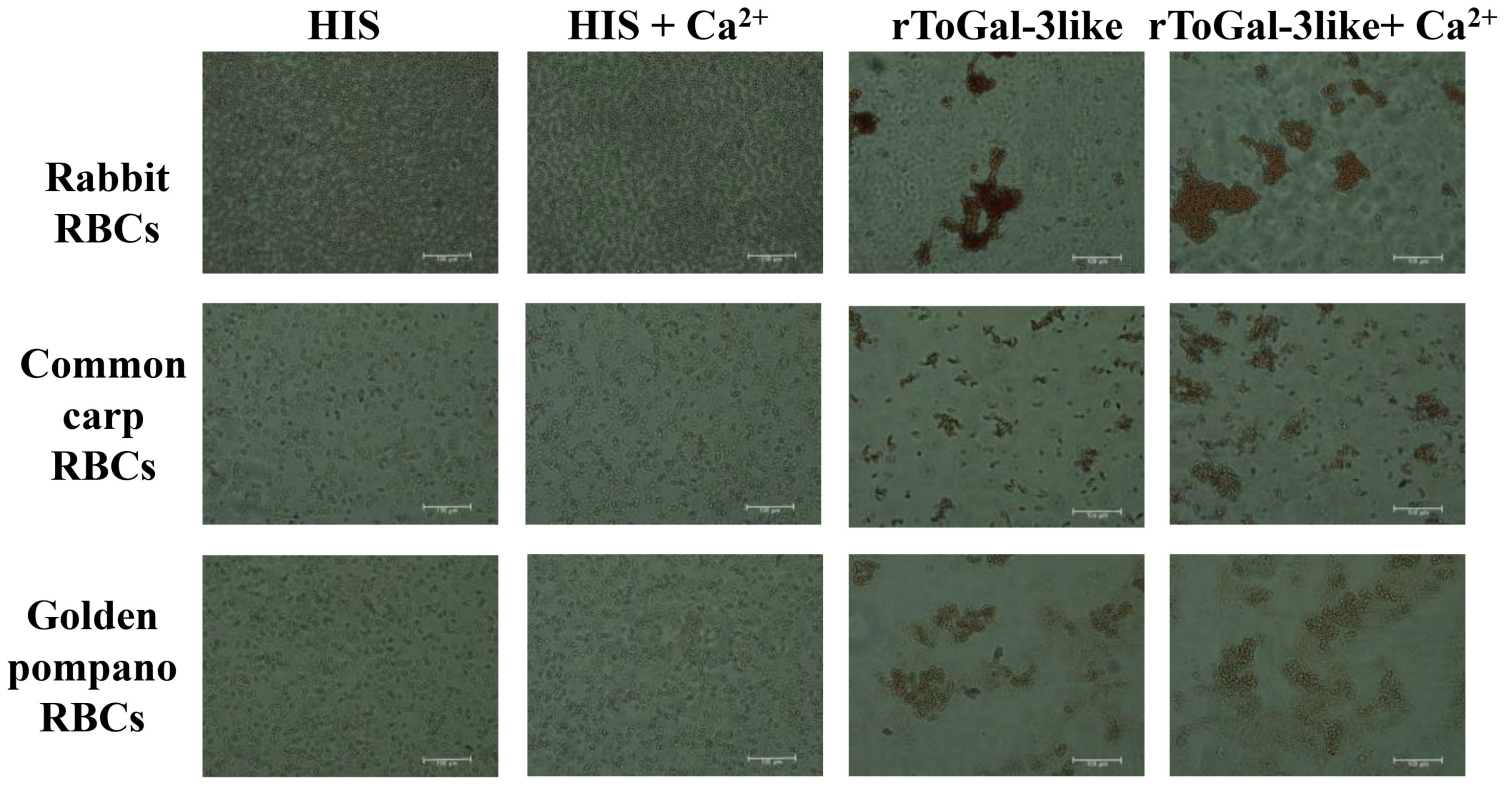
Hemagglutination of rToGal-3like. RBCs from rabbit, Common Carp, and Golden pompano have been agglutination by rToGal-3like. The His protein groups were as negative controls.

The 2% RBC of rabbit, carp, and golden pompano were prepared using the centrifugation and resuspension method. The rToGal-3like was added to observe hemagglutination. The hemagglutination assay demonstrated that rToGal-3like could hemagglutination the RBC of rabbit, carp, and golden pompano, and this activity was independent of Ca^2+^ presence (**Figure 8**). In the control group, under both the presence and absence of Ca^2+^, there was no agglutination of RBC from any of the three species. Thus, ToGal-3like possesses agglutinating activity of RBC.

### 3.10 Bacterial agglutination and antibacterial activity of rToGal-3like

**Figure 9:**
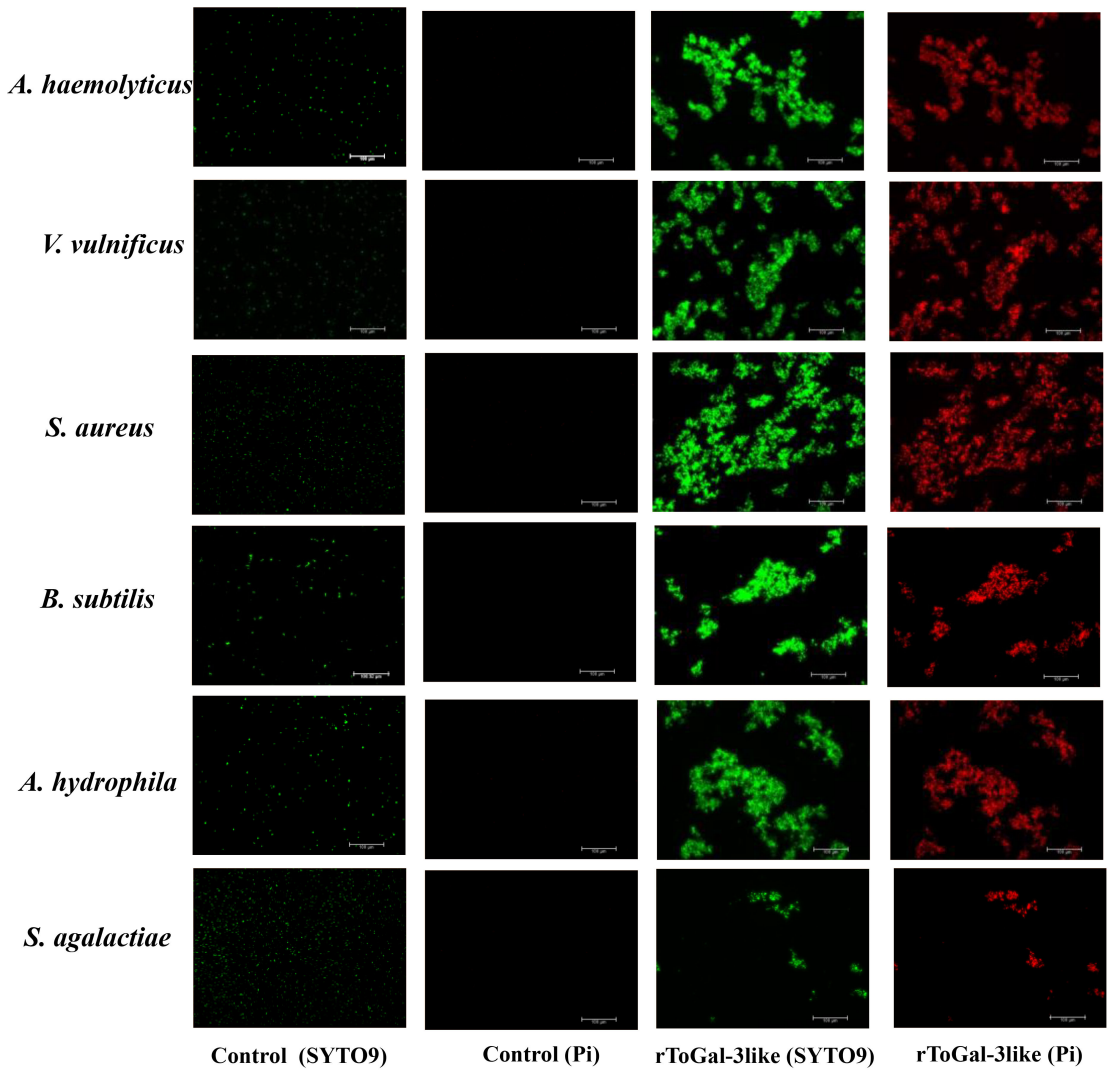
Bacterial agglutinations by rToGal-3like. All the bacteria stained by SYTO 9 were in green and dead bacteria stained by Pi were in red. Agglutination occurred in *S. aureus, B subtilis, V. vulnificus, S. agalactiae, A haemolyticus* and *A hydrophila*.

In this study, bacterial agglutination assays with rToGal-3like were conducted using four Gram-negative bacteria: *V. vulnificus, S. agalactiae, P. aeruginosa* and *A. hydrophila*, as well as two Gram-positive bacteria: *S. aureus* and *B. subtilis* (**Figure 9**). It was observed that in the control group under SYTO9 imaging, bacteria remained agglomerated, appearing as scattered green fluorescence. In contrast, in the test group treated with rToGal-3like, clear bacterial agglutination was observable under SYTO9 imaging, and most bacteria emitted red fluorescence when stained with Pi. rToGal-3like demonstrated the ability to agglutinate and kill both Gram-negative and Gram-positive bacteria.

## 4 Discussion

This study analyzed the number of *Gals* members in the genomes of three teleost fish—golden pompano, turbot, and zebrafish—and two mammals—humans and mice—as well as chickens. The number of *ToGals* was similar across species, but *Gal-6* and *Gal-7* were absent in teleost fish. Gal-6 has also been identified in insects such as the Egyptian mosquito, where it has been found to block the binding of Cry11Aa in the widely used agricultural insecticide BT to ALP1 (Alkaline Phosphatase 1) and APN2 (Aminopeptidase N2), thereby neutralizing the toxicity of the insecticide (25). *Gal-7* was first discovered about 30 years ago in epithelial tissues involved in apoptotic responses (26). Their functions are similar to those of other *Galectins*, which may be the reason for their evolutionary loss (27).

The prediction of conserved domains in ToGals revealed that the tandem repeat *ToGals*, such as *ToGal-8*, *ToGal-9* and *ToGal-4*, have two CRDs, but the conserved motifs corresponding to these homologous CRDs are not identical. They are connected by a hinge region, and the coordination between the two CRDs and the linker region is crucial for the protein’s function. According to studies by Hsu et al (28) and Levy et al. (29). the C-terminal CRD tends to bind with hydrophilic glycoproteins, while the N-terminal CRD more often associates with hydrophobic glycoproteins or glycolipids, a characteristic that appears to be common among all isotypes. This finding is also supported by the research of Ideo et al (30). Furthermore, Yoshida et al. discovered that the C-terminal and N-terminal CRDs respectively show higher binding affinity to oligosaccharides on N-glycan branches and to sugars that are 3′-O-sulfated or 3′-O-sialylated (31). These functional differences are likely a significant reason for the variations in the conserved motifs between the two CRDs.

This study also analyzed the expression patterns of *ToGals* following an *S. agalactiae* infection. Initially, *ToGal-3* expression in the spleen and liver was upregulated and subsequently downregulated, mirroring the response seen in grass carp injected with GCRV and various PAMPs, where *Gal-3* expression notably increased before decreasing (Zhu et al., 2020). Moreover, *Gal-3*’s role in antimicrobial immune responses includes pathogen recognition and regulation of monocytes/macrophages’ activity (32). Similarly, post-infection, *ToGal-2a* expression rose in the spleen and kidneys, a pattern also observed in *Sinonovacula constricta* and *S.maximus* after *Vibrio anguillarum* infection. Additionally, following the injection of rOnGal-2 in Nile tilapia, both antioxidant and antibacterial activities were enhanced (33). These observations underscore the critical role of *Gals i*n antimicrobial processes.

Following infection with *C. irritans*, the various members of *ToGals* exhibited distinct expression patterns. Notably, although ToGal-3like showed overall lower expression levels compared to *ToGal-3* at all sites, it consistently displayed lower expression in TAS compared to NRS and BFS. Gal-3 has been identified to play a role as a PRR in humans infected with *Leishmania amazonensis*, controlling parasite invasion, proliferation, and the formation of phagosomes (34). The absence of *Gal-3* is known to enhance intracellular parasite replication *in vitro*, increase systemic parasitemia *in vivo*, and decrease the recruitment of leukocytes, with observations showing reduced secretion of pro-inflammatory cytokines in the spleens and hearts of infected *Gal-3* knockout mice (35). ToGal-9 was found to have the highest expression in TAS. While there is limited research on the role of *Galectin-9* in teleost fish following parasitic infections, studies have found that in mice, the expression of *Tim-3/Galectin-9* is upregulated following malaria-induced acute lung injury, where Gal-9 plays a crucial immunoregulatory role by inducing apoptosis or inhibiting effector functions through binding to its receptor Tim-3 (36).

The subcellular localization results indicate that ToGal-3like is distributed in both the cytoplasm and nucleus of GPM. Numerous observations have been reported on the distribution of Gal-3 in the nucleus and cytoplasm (37, 38). The COOH-terminal end of Gal-3 (the last 28 aa) is crucial for nuclear localization, Gal-3 shuttles between the cytoplasm and nucleus based on targeting signals recognized by importins for nuclear localization and exportin-1 (CRM1) for nuclear export (39). In fact, many ligands of Gal-3 in both the cytoplasm and nucleus have been reported. For example, in the cytoplasm, Gal-3 interacts with the apoptosis inhibitor (Bcl-2), and this interaction may involve the anti-apoptotic activity of Gal-3 (40). In the nucleus, Gal-3 is an essential precursor mRNA splicing factor; the protein integrates into the spliceosome by binding to the U1 small nuclear ribonucleoprotein (snRNP) complex (41). Thus, ToGal-3 plays important roles in both the nucleus and cytoplasm.

Furthermore, Galectins also function in the extracellular matrix, where bacterial agglutination is another vital defensive action against invading pathogens. Galectins can trap pathogens in the extracellular matrix before they enter host cells, which may then facilitate the phagocytic action of macrophages (42). In this study, four Gram-negative and two Gram-positive bacteria underwent agglutination under the influence of rToGal-3like. Previous research has shown that the recombinant protein of Gal-3 in Nile tilapia can agglutinate *S. agalactiae* and *A. hydrophila* (32). Lipopolysaccharide (LPS) is the primary PAMP in Gram-negative bacteria and a major ligand for galectins. LPS consists of three main components: lipid A, core polysaccharide, and O antigen (43), with galectins primarily interacting with the β-galactoside polysaccharides in the O-antigen region. Although Gram-positive bacteria lack β-galactoside polysaccharides, Gals can bind to hydrophobic ligands in the peptidoglycan (PGN) of the cell wall (44, 45). This might explain why rToGal-3like showed agglutination/binding activity with both Gram-positive and Gram-negative bacteria in this study. Most importantly, this foreshadowed the potential function of ToGal-3like as a PRR in innate immunity.

## 5 Conclusion

This study systematically identified and characterized 10 *ToGals* genes in golden pompano. Their motifs, protein domains, and collinearity relationships were analyzed. The regulation of *ToGals* following infection with *S. agalactiae* and *C. irritans* indicates their involvement in immune responses against parasites and bacteria. A key member, *ToGal-3like*, was selected for further analysis. Subcellular localization results showed that ToGal-3like protein is expressed in both the cytoplasm and nucleus. Finally, the recombinant rToGal-3like was obtained through prokaryotic expression. rToGal-3like has the ability to agglutinate various RBCs without the need for Ca^2+^ and can also agglutinate various Gram-negative and Gram-positive bacteria. This study lays a scientific foundation for further research on the function of *Gals* in teleost, particularly highlighting the potential role of Gals as PRRs in innate immunity. However, more experimental data are needed to reveal or validate the findings of this study.

## Data availability statement

The datasets presented in this study can be found in online repositories. The names of the repository/repositories and accession number(s) can be found below: https://www.ncbi.nlm.nih.gov/genbank/, GCA_900607315.1.

## Ethics statement

The animal study was approved by The Committee of the South China Sea Fisheries Research Institute, Chinese Academy of Fisheries Sciences. The study was conducted in accordance with the local legislation and institutional requirements.

## Author contributions

J-MP: Data curation, Formal analysis, Methodology, Visualization, Writing – original draft, Writing – review & editing. YL: Writing – original draft, Writing – review & editing. K-CZ: Methodology, Writing – review & editing. H-YG: Conceptualization, Writing – review & editing. B-SL: Methodology, Writing – review & editing. NZ: Conceptualization, Writing – review & editing. LX: Software, Writing – review & editing. T-FZ: Software, Writing – review & editing. D-CZ: Conceptualization, Writing – original draft.
