# Supplementary material for: Genome-wide characterization, phylogenetic and expression analysis of Galectin gene family in Golden pompano Trachinotus ovatus
Source: Front Immunol. 2024 Jul 18;15:1452609. doi: 10.3389/fimmu.2024.1452609 (PMC11291232; doi:10.3389/fimmu.2024.1452609)
Supplement: Supplementary file 1 [file DataSheet_1.docx]

**Figure. S1. SDS-PAGE detection** Note: A, SDS-PAGE gel of total bacterial protein from positive strains, Lane M: Protein molecular weight standard (116.0/66.2/45.0/35.0/25.0/18.4/14.4 kDa), Lane 1: Control, Lane 2: IPTG-induced total bacterial protein. B, SDS-PAGE gel of the supernatant and precipitate from the cell lysate of bacteria with high-level expression, Lane M: Protein molecular weight standard, Lane 1: Supernatant protein after induction and lysis, Lane 2: Insoluble protein after induction and lysis. C, SDS-PAGE gel of eluted concentrated protein, Lane M: Protein molecular weight standard, Lane 1: Eluted concentrated protein.

**Figure S1**
